# Current Status of the Regulation for Medical Devices

**DOI:** 10.4103/0250-474X.49085

**Published:** 2008

**Authors:** Anuja R. Shah, R. K. Goyal

**Affiliations:** L. M. College of Pharmacy, P.O. Box 4011, Navrangpura, Ahmedabad-380 009, India

**Keywords:** Drugs and cosmetics act, medical devices, regulations, role of pharmacists

## Abstract

In the light of escalating use of medical devices, stringent regulatory standards are required to ensure that the devices are safe, well studied and have least adverse reactions. Recently introduced guidelines and the amendment in the law will provide adequate guidance for both the manufacturers and competent authorities to manage cases efficiently and appropriately. India has emerged as one of the leaders in pharmaceutical industry. Like many other amendments in Drugs and Cosmetics Act that have boosted the global confidence in pharmaceutical industry in India, guidelines for devices will encourage the much needed research in medical devices. Pharmacy personnel can certainly play an important role in the regulation of medical devices. Safety, risks, effectiveness and performance of the medial devices need to be well established and regulated properly. It is hoped that the guidelines are implemented and regulated properly with effective outcome.

On October 6, 2005, the Government of India released the Gazette indicating sterile devices as drugs (F. No. 11014/2/2005—DMS and PFA; Gazette No. 1077 dated October 6, 2005) under the sub-clause (iv) of clause (b) of section 3 of Drugs and Cosmetics Act 1940 (23 of 1946)^1^. Earlier as per the sub-clause (iv) of the clause (b) of section 3 of Drugs and Cosmetics Act 1940 (23 of 1946) the definition of drugs included the items “such devices intended for internal or external use in the diagnosis, treatment, mitigation or prevention of disease or disorder in human beings or animals” as may be specified from time to time by the Central Government by notification in the Official Gazette, after consultation with the Board^2^. With this notification various items have been specified as drugs, as given in Table 1.

**TABLE 1:** GUIDELINES FOR THE IMPORT AND MANUFACTURE OF MEDICAL DEVICES^1^,^2^

Categories	Examples and explanations
1. Sterile devices considered as drugs under Section 3 (b) (iv) of the Act.	Cardiac stents Drug eluting stents Catheters Intra ocular Lenses LV cannulae Bone cements Heart valves Scalp vein Set Orthopedic implants Internal prosthetic replacements
2. Period for the import of medical devices	A period of 60 days would be provided for the importers to make application for import and registration from the date of publication of these guidelines.
3. Details required for the registration of medical devices for the import	Applicant details Product information Regulatory status Master file (Details of Good Manufacturing Practices employed by the manufacturer to ensure quality of the device) Devices containing medicinal product Post market surveillance Undertaking of conformity with respect to product standards, safety and effectiveness requirements and quality systems in the country of origin.
4. Details required for the license to manufacture of Medical Devices in the Country	Manufacturing details Product details

It has been further notified vide GSR 127 (E) dated 7/10/2005 that the control over manufacture of these devices would be exercised by CLAA i.e. DCG (I) under the provisions of sub-rule (1) of rule 68A of part VII of the Drugs and Cosmetics Rule, 1945. These rules have been approved by the Ministry of Health and Family welfare and the guidelines issued came in force from March 1, 2006. Some of the highlights of these guidelines are given in Table 1.

## Significance of Medical Devices:

The era of newer development and technology has decreased the morbidity and mortality of life. The medical development in terms of drugs and devices has brought about the robust change in the life of the people (as offered by the cosmetic treatment, dentist, face and cardiology devices). Medical devices have extended the ability of physicians to diagnose and treat diseases, making great contributions to health and quality of life.

According to World Health Organization (Geneva), under Medical Device Regulations, the term “medical devices” includes everything from highly sophisticated computerized medical equipment down to simple wooden tongue depressors. The intended primary mode of action of a medical device on the human body, in contrast with that of medicinal products, is not metabolic, immunological or pharmacological. Medical devices include a wide range of products such as medical gloves, bandages, syringes, condoms, contact lenses, disinfectants, X-ray equipment, surgical lasers, pacemakers, dialysis equipment, baby incubators and heart valves. Medical device means any instrument, apparatus, implant, machine, appliance, implant, *in vitro* reagent or calibrator, software, material or other similar or related articles, intended by the manufacturer to be used, alone or in combination, for human beings for one or more of the specific purposes like diagnosis, prevention, monitoring, treatment or alleviation of disease; diagnosis, monitoring, treatment, alleviation of or compensation for an injury; investigation, replacement, modification or support of the anatomy or of a physiological process; supporting or sustaining life; control of conception; disinfection of medical devices and providing information for medical purposes by means of *in vitro* examination of specimens derived from the human body and does not achieve its primary intended action in or on the human body by pharmacological, immunological or metabolic means, but may be assisted in its function by such means^3^.

Medical devices are now a pervasive part of modern medical care. They are in many cases associated with quality of care. In some cases, the use of devices has certainly improved quality. In other cases, devices have associated with many problems. The approach to quality of devices has depended largely on regulation. According to global statistics, 85% of the medical devices are manufactured in the USA, in Japan and in European Union countries. That is the reason why it is matter of concerns to the American and European regulation systems^3^.

Like medicines and other health technologies, they are essential for patient care at the bedside, at the rural health clinics or at the large, specialized hospitals. Medical devices also add to the financial burden on the Government health sector. The medical devices market is showing a double-digit growth. The cardiac devices alone are growing at 20 per cent. In India, the growth of the market is estimated to be between 10-15 per cent. There is a clear indication that the penetration levels are higher in the country. This is because of affordability by patients, increased awareness on health care, improved hospital infrastructure and the increased disease patterns^4^.

The public expects that medical devices meet the highest safety standards. Realizing the importance of Pharmacovigilance, Ministry of Health and Family Welfare, Government of India, with WHO funding, initiated a country wide National Pharmacovigilance Program. Central Drugs Standard Control Organization (CDSCO), New Delhi, coordinates the program. The Honorable Minister of Health, Dr. Anbumani Ramadass at New Delhi, officially launched the program on November 23, 2004. CDSCO has established 2 zonal centres, 5 regional centres and 28 peripheral centres all over India^4^.

## Classification of medical devices from regulatory view point:

Medical devices may be classified as per their medical utility or technical design and manufacturing aspects. However, regulatory authorities around the world have classified them based on their safety requirements and standards of quality to be set. Several criteria are considered to evaluate the potential risk: degree of invasiveness, duration of contact, affected body system and local versus systemic effects. The classification of medical devices differs from country to country but the classification in Table 2 gives a comprehensive view of various classes of medical devices.

**TABLE 2:** COMPREHENSIVE CLASSIFICATION OF MEDICAL DEVICES^3^

Europe	US FDA	GHTF (Japan)	Examples of included medical devices
Class I	Class I*	Class A	Non sterile items or sterile items with a low potential risk: surgical instruments, urine bags, stethoscope, examination gloves
Class IIa	Class II	Class B	Sterile items surgical gloves, urinary catheters, stomach tubes, needles, tracheal tubes, IV giving sets
Class IIb	Class II	Class C	Blood bags, condoms, non-absorbable sutures, anaesthesia machines
Class III	Class III	Class D	Absorbables sutures

* With or without GMP

In Europe medical devices must comply with the requirements of the Directive, in accordance with the existing European Norms, and with the monographs of the European Pharmacopoeia (for sutures). The European Norms are edited by the European Committee for Normalization. The national bodies of European Committee for Normalization convert these norms into their respective national standards within 6 months following the publication. The regulatory requirement for different classes of medical devices is given in Table 3^5^.

**TABLE 3:** REGULATORY REQUIREMENTS UNDER EUROPEAN COMMITTEE FOR NORMALIZATION^5^

Category	Explanation
Class 1	The conformity assessment procedures can be carried out under the sole responsibility of the manufacturer (low potential risk), except for sterile devices or devices with a manufacturing function. In this case, the intervention of a notified body is necessary.
Class IIa	The intervention of a notified body is compulsory at the production stage.
Class IIb	The intervention of a notified body is compulsory to control the design and the manufacture.
Class III	The intervention of a notified body is compulsory to control the design and the manufacture. An explicit prior authorization with regard to conformity is also required.

According to the Directive 93/42/CEE and the US regulation 21 CFR 820, in the USA, the procedure to obtain an accreditation depends on the classification of the medical device. The marketing of a medical devices is a subject to the FDA controls and unless exempt require “A marketing clearance”. Table 4 gives regulatory requirements in US for medical devices^6^. Australia registers for therapeutic goods in Australia has classified the medical devices into five classes (Table 5). The placement of *in vitro* diagnostic medical devices in the new system is still under consideration. All classes are required to demonstrate conformity with safety and performance requirements. Class IIa, IIb, III and Active implantable medical devices (AIMD) require quality systems verification. Class III devices and AIMDs are subject to the most extensive pre-market assessments^7^.

**TABLE 4:** REGULATORY REQUIREMENTS IN THE UNITED STATES FOR MEDICAL DEVICES^6^

Category	Explanation
Class I	Most class I devices are exempted from clearance, but they are subject to the general control requirements
Class II	Most class II and some class I devices require a marketing clearance of which the obtaining is subject to the 510(k procedure. To get it, the manufacturer must submit to the FDA an information pack which shows that the proposed device is substantially equivalent to an already existing device on the American market.
Class III	Most class III devices and new devices require a marketing clearance of which the obtaining procedures (Pre-Market Approval [PMA] or Product Development Protocol [PDP]) are more stringent than the 510 (k).

**TABLE 5:** CLASSIFICATION FOR MEDICAL DEVICES BY AUSTRALIAN REGISTER FOR THERAPEUTIC GOODS^7^

Category	Explanation
Class I	Low risk: These devices are non-invasive and simple or invasive and transient use/reusable, e.g., non-powered hospital furniture, some devices, classified “listable” such as stethoscopes, examination gloves, dentures, wound dressings and simple surgical implements like non-sterile, non-powered surgical and dental instruments
Class II	Intermediate risk: Devices in this class are subdivided into Class IIa and Class IIb, depending on the level of invasiveness
Class IIa	devices are non-invasive for channeling or body fluid modifiers; special dressing; invasive and short term use; diagnostic active; device hospital and household/ commercial grade disinfectants, e.g., infusion tubing, polymer film dressing, urinary catheters, suture needles, hearing aids, dental filling materials and oxygen meters
Class IIb	devices include ‘healing’ wound dressings; surgically invasive; implantable; active; contraceptives; blood bags, e.g., hemodialysers, insulin pens, bone cement, intra-ocular lenses, anaesthetic machines
Class III	High risk: These devices include the surgically invasive, including medicine and animal-derived products, e.g., all active implantable devices, cardiovascular catheters, absorbable sutures, heart valves and collagen implants
AIMD	Active implantable medical devices, equivalent to in risk to Class III

## Regulation of devices:

The approach to quality of devices depends largely on regulation. In addition, there are many problems in the interface between the machine and the user or the patient that are largely untouched by device regulation, and are considered in quality assurance programs. As essential as device regulation is, it is not sufficient to assure quality. Education is particularly important in this area. Quality assurance programs need to be familiar with common problems with medical devices and how to approach them.

The regulation of medical devices is a vast and rapidly evolving field that is often complicated by legal technicalities. For example, legal terms and their meanings are sometimes non-uniform even within one regulatory system. Optimum safety and performance require among all involved in the life span of a medical device: the government, the manufacturer, the importer/vendor, the user and the public each has a specific role to play in this risk management.

## Regulation of medical devices in some countries:

The regulations (or standards, or norms) are intended to protect the user against the risks associated with design, manufacture and packaging of medical devices. They differ from one country to another.

As a science-based regulatory agency, the US Food and Drug Administration (FDA), is responsible for a large and diverse array of products. Since 1976, that responsibility has included insuring the safety and effectiveness of medical devices. The universe of these medical devices is immense, including approximately 5,000 different types of products encompassing a spectrum of technologies from microelectronics to microbiology. FDA's Center for Devices and Radiological Health (CDRH) regularly monitors trends that point toward future product development. The manufacture and quality assurance of medical devices in USA is subject to the regulation 21 CFR 820 (or Quality System Regulation) and its audit reports are publicly accessible. This regulation came into force in 1978 (20 years earlier than the EU Directive). The US FDA registers the product and authorizes the manufacturer to market it in USA. All the technical monographs published by the profession (associations of manufacturers), as well as the profession's practices have legal force. The US FDA is a single body is being imposed by the national authorities. The inspections are led by sworn inspectors. The competence level is very high. Sanctions are possible in case of non-compliance to the regulation^6^,^8^–^13^.

The Australian medical devices industry plays an important role in Australia's health sector. Australia is among the world leaders in ensuring high standard international regulation and is one of the five members of the Global Harmonization Task Force (GHTF) for medical devices along with the US, Canada, the European Union and Japan. The GHTF publishes guidelines on basic regulatory practices, but there is nothing concerning the application of these guidelines and no proper inspection is carried out^3^. In Europe three laws, referred to as directives, directive 93/42/EEC: medical devices; directive 90/385/EEC: active implantable medical devices; and directive 98/79/EEC: *in vitro* diagnostic medical devices are in force in the European Union countries. Only the 93/42/EEC directive concerns the medical equipment which came into effect on June 14, 1998 although the project was published on June 14, 1993. The manufacturers have to meet the requirements of this directive to get the CE marking on a medical device. This marking is mandatory for the marketing and the free circulation of the medical devices in the EU countries without additional control or administrative procedure. This directive also applies to subcontractors. The laws enforcement (Directive 93/42/EEC) is controlled by national bodies or notified bodies whose audits reports are publicly assessable^5^.

The new ISO 13485 (2003) standard, specific to medical devices, replaces the ISO 9001 (2000) generic standard. They contain technical specifications or other precise criteria, which have to be used coherently as rules, guidelines or definitions to ensure that materials, products, processes and services are fit to their purpose. The standards related to the quality assurance system are grouped in the ISO 9000 family. They are generic standards, i.e. their requirements apply to any company, whatever the manufactured product or the delivered service. The ISO 9001 (2000) standard covers the whole system of activities starting from the conception until the sale of the article. It has become the international reference standard for the quality assurance system of medical devices, and even if it is not mandatory. It gets practically a legal force in Europe. Various bodies are appointed by each member state of the EU (Ministry of Health, Ministry of Industry and so on), which has to inform the European Commission, and the other member state of it. The European Commission publishes the list (regularly updated) of the notified bodies, together with their identification number (4 numbers following the CE marking) and the defined tasks for which they have been notified. To carry out the certification of conformity procedures, the manufacturer may apply to the notified body of his choice in any EU country. In practice, the quality level strongly varies from one notified body to another^14^.

Developing countries usually do not have their own regulations on medical devices, but many of them refer to the European or American normative system, including GHTF to facilitate the sell of their products in Europe and USA. Since medical devices caused some accidents, sometimes fatal, their manufacturing process must comply with the Good Manufacturing Practices (GMP). There is a very strict quality assurance on all aspects of the production of the medical devices in order to protect the patient's health. In 1969 the GMP standards were drawn by the WHO for drugs, in 1976 it included regulation 21 CFR 211 on drugs in the USA, and in 1997 it included the regulation 21 CFR 820 on medical devices in the USA^6^.

In India the major source of pharmaceutical regulations is the Drugs and Cosmetics Act 1940. This legislation applies to the whole of India and for all products whether indigenous or imported. The legislation is enforced by the office of the Drugs Controller General of India (DCGI). However, at the field level, enforcement is done by the individual state Governments through their Food and Drug Control Administration (FDCA). Matters of product approval standards, clinical trials introduction of new drugs, and import license for new drugs are handled by the DCGI. With the help of Indian Council of Medical Research, New Delhi the approvals for setting up manufacturing facilities and obtaining license to sell and stock drugs are provided by the State Government. However, a similarly regulatory body for the Medical Devices rules and regulations is yet to be established properly.

## Role of Pharmacist in regulation on use of devices:

India has emerged as one of the leaders in pharmaceutical industry. The Indian Pharma sector is growing exponentially. Its value in 2004 was US$ 6 billion which has increased to US$ 10 billion at the end of year 2006. On the manufacturing side there are 23 000 manufacturers (1.2% in formulation, the rest in bulk drugs), imports are 4% of total size of domestic market value US $ 3 billion, and export is Rs. 30 000 crore (2007-08)^15^. Indian drug prices are among the lowest in the world. India has recently being viewed as a place with great potential for clinical research. The pharmaceutical sector and especially the pharmacists have been playing a lead role in these directions. Medical device sector has so far not been even in the thought process of pharmaceutical sector. Pharmacy graduates or post-graduates are not even aware of aware of various medical devices used in hospitals. There are very few pharmaceutical companies that have taken a lead in medical devices (except syringes, medical gloves, bandages, condoms, contact lens, disinfectants, etc). Pharmacy personnel can certainly play an important role in the regulation of medical devices. Following are the steps needed to play a positive role in the reputation of medical devices though pharmacists^3^.

It is necessary to have proper understanding of medical device safety, risk involved, the degree of invasiveness, duration of contact, the body system affected, and local versus systemic effects. One should weigh the risks against the benefits to the patients compatible with a high level of protection of health and safety so that maximum benefit and minimum risk is ensure when a device is being used by doctor.

Pharmacist should be actively involved in the regulation of effectiveness and performance of medical device. One has to provide clinically effective parameters through the manufacturer which are relative to the medical condition. Clinical effectiveness is a good indicator of device performance, which is closely linked to safety.

Pharmacists should be involved in the documentary of standards containing technical specifications or other precise criteria to be used consistently as rules, guidelines or definitions of characteristics, to ensure that materials, products, process and services are fit for their purpose. One has to ensure that the prescriptive, design, performance, and management specifications meet the standards.

Pharmacists can promote to establish voluntary standards by consensus from all interested parties (the stakeholders). The use of voluntary/consensus standards may be developed by experts with access to the vast resources available in the professional and industrial communities. Conformity to such standards can also be assessed by an accredited third party (such as a notified body in Europe), and thereby improve and update the standards. All these can make medical device standards effective and efficient tools for supporting health care, and provide to the manufacturers have the flexibility to choose appropriate standards or other means to demonstrate compliance with regulatory requirements.

Many countries lack access to high-quality devices and equipments that are appropriate for their specific epidemiological needs. This is particularly true in developing countries, where health technology assessments are rare and where little regulatory controls exist to prevent the import or use of substandard devices. With the vast majority of devices in developing countries being imported and this may increase the risk and need to be considered lives at risk.

## CONCLUSIONS

In the era of newer research and development, technology may have both curse and bless for the lives of human beings. Hence, a proper and stringent rules and regulations need to be put forth in the practice. Different regulatory bodies exist that regulate or monitor the activities undergoing in terms of both socio-economic protection of human beings. Looking to scope and requirement of medical devices, India needs to enter in the global market to manufacture their own devices. Thus, a proper rules and regulations are needed to encourage the efficient growth of device industry.

Rules and Regulations for medical devices are required in India. Since, the world market is seeing the accentuating use of medical devices in varied type of patients and with unique patterns of disease, this will not only give a public safety assurance but also the manufacturer will get a detailed, accurate, long term surveillance of the medical device, generating more information and hints for further improvements. Education is particularly important in this area. Quality assurance programs need to be familiar with common problems with medical devices and how to approach them.
